# Factors influencing healthcare professionals’ rating on quality of death and dying: a nationwide cross-sectional study in China

**DOI:** 10.3389/fpubh.2025.1722430

**Published:** 2025-11-20

**Authors:** Mingming Cheng, Yaxin Lan, Yongyi Chen, Fei Fang

**Affiliations:** 1 Department of Social Work, School of Sociology and Political Science, Shanghai University, Shanghai, China; 2 Hunan Cancer Hospital/The Affiliated Cancer Hospital of Xiangya School of Medicine, Central South University, Changsha, Hunan, China; 3 Department of Sociology, School of Sociology and Political Science, Shanghai University, Shanghai, China

**Keywords:** cross-sectional study, palliative care, hospice, quality of death, education, healthcare professionals

## Abstract

Understanding healthcare professionals’ perspectives on the quality of death and dying is essential for improving palliative care in China, especially as hospice and palliative services expand. This study examined how Chinese healthcare professionals’ assessments of death and dying quality vary by institutional setting, sociodemographic factors, professional background, and training experience. A cross-sectional design was conducted. Between November 2023 and January 2024, 2,465 healthcare professionals engaged in palliative and hospice care (including doctors, nurses, and social workers) participated in the survey. Multivariate linear regression models and propensity score matching were used to identify factors associated with Quality of Death and Dying Index ratings. Higher ratings on the Index were observed among respondents working in institutions with multidisciplinary end-of-life care teams, those who were female, born in the 1970s, held a bachelor’s degree, had longer years of service, cared for patients with a survival period of three to six months, and participated in palliative care training programs. These findings suggest that institutional collaboration, professional experience, and targeted training are closely linked to more favorable perceptions of death and dying quality among healthcare workers. Enhancing multidisciplinary coordination and ensuring equitable access to training opportunities may help improve end-of-life care quality within healthcare institutions. The results provide evidence-based insights into the determinants of death and dying quality in China, offering practical guidance for strengthening palliative and hospice care development in rapidly aging societies where end-of-life service systems are still evolving.

## 1 Introduction

The quality of death and dying is a multidimensional construct reflecting individuals’ physical, psychological, social, and spiritual experiences in their final stage of life (1). It serves as a critical benchmark for evaluating hospice and palliative care, capturing not only the quality of life before death but also the effectiveness of hospice and palliative interventions (2, 3). Healthcare providers’ evaluations offer a complementary perspective to those of patients and families, providing systematic and expert-based insights into the strengths and weaknesses of current end-of-life care practices (4, 5). Professional assessments are vital for identifying areas of improvement, informing policy decisions, and advancing the quality of hospice and palliative care (6, 7).

In 2021, research teams supported by the Lien Foundation developed the Quality of Death and Dying Index (QODDI), a 13-item instrument assessing critical domains of EOL experience, including care environment, communication, medical treatment, pain management, and financial burden (8). This index has since been applied in international comparisons, revealing persistent disparities across countries (7, 9). Mainland China ranked 71st in 2015 and 53rd in 2021, highlighting ongoing challenges in providing accessible, high-quality palliative and hospice care (7, 10).

Palliative and hospice care in China remains at an early stage of development, characterized by considerable regional disparities in service accessibility and quality (11). Variations in healthcare infrastructure and resources between regions contribute significantly to these gaps, especially in economically disadvantaged areas, where the integration of palliative care into the national healthcare system remains limited (12, 13). Moreover, access to hospice services is often inadequate, and standardized protocols for end-of-life care are not yet fully established (14, 15). These challenges are further exacerbated by persistent cultural stigmas surrounding death and dying, which continue to hinder open discussions and the wider adoption of palliative services (16, 17). Consequently, healthcare professionals’ evaluations of end-of-life quality may vary substantially, reflecting differences in personal attributes, professional experience, and institutional settings.

Improving the quality of death and dying has become increasingly urgent in China amid rapid population aging. The *Healthy China 2030* initiative emphasizes the integration of health and senior care services and calls for a coordinated, efficient, and equitable healthcare system. Within this framework, the policy explicitly prioritizes the development of palliative and hospice care alongside long-term care and rehabilitation, promoting accessible end-of-life services across urban and rural areas (18). Since 2017, the National Health Commission has launched three nationwide pilot projects aimed at expanding palliative care services, initially focusing on specific urban areas and now covering 185 cities and districts. These efforts have prompted local governments to implement supportive policies for healthcare providers (19, 20). However, formal education on hospice and palliative care remains limited in most medical schools in China (21, 22), prompting reliance on continuing education and in-service training to address workforce shortages (23, 24). The effectiveness of these training programs, however, has yet to be systematically evaluated.

To date, empirical research exploring these inconsistencies in professional assessments is still limited. Few studies have systematically examined how demographic, institutional, and training-related factors shape healthcare providers’ perceptions of death and dying quality in China. Furthermore, the impact of palliative care training programs on professionals’ evaluations has not been empirically tested on a national scale.

To address these gaps, this study conducted a large-scale national survey among healthcare professionals engaged in hospice and palliative care. It aimed to identify demographic and institutional determinants of QODDI evaluations and to examine the effects of palliative care training using propensity score matching, a quasi-experimental approach. The findings of this study have the potential to inform policy and practice improvements, contributing to the development of more equitable and high-quality end-of-life care in the Chinese context.

## 2 Materials and methods

### 2.1 Participants and sampling

This study utilized a cross-sectional survey design, employing a structured online questionnaire to collect data. Prior to the commencement of data collection, the research protocol received ethical approval from the Science and Ethics Committee of Shanghai University, with the approval number [ECSHU2022-220]. The study adhered to the STROBE guidelines for cross-sectional studies. Written informed consents were provided, and all respondents volunteered to participate.

The survey was conducted between November 2023 and January 2024 using a convenience sampling approach through two recruitment networks. Nationwide data were gathered in collaboration with the Palliative and Hospice Care Committee of the China Anti-Cancer Association. Under the coordination of the committee’s secretariat, invitations were distributed to registered member institutions providing hospice or palliative care services, including oncology departments and palliative care units. Institutional representatives were invited to complete an online questionnaire. In parallel, data collection in Shanghai targeted institutions with formally established palliative care departments, given the city’s advanced specialization in end-of-life services. The research team contacted hospital directors and palliative care leaders, who distributed the same online survey link to healthcare professionals involved in end-of-life care within their institutions. These recruitment channels allowed broad coverage across institutional types and regions, facilitating high participation rates and ensuring adequate response numbers for robust analysis.

Participation was entirely voluntary. Eligible respondents included physicians, nurses, and medical social workers with experience in providing palliative or hospice care. A total of 3,018 responses were obtained, of which 2,465 were deemed valid for analysis. These responses originated from 1874 medical institutions, including both public and private entities involved in palliative and hospice care provision. The sample was drawn from all 30 provinces and municipalities of Mainland China, ensuring broad geographic coverage. The sample selection process figure is depicted in Supplementary Figure 1.

To ensure confidentiality, data collection excluded personally identifiable information, and all responses were anonymized before analysis. Personnel involved in data handling and management signed confidentiality agreements. The anonymized dataset is available upon reasonable request from the corresponding authors.

### 2.2 Measures

#### 2.2.1 Quality of death and dying index

The Quality of Death and Dying Index was derived from a 13-item scale designed by the Lien Foundation in 2021. This scale encompasses various aspects of end-of-life care, including the hospital environment, physician-patient communication, medical treatment, pain management, and cost considerations. Each item was rated on a five-point Likert scale, ranging from “strongly disagree” to “strongly agree.”

Following the developers’ recommendation, preference weights derived from the discrete choice experiment conducted by the original research team were applied to compute the total QODDI score. Researchers quantified the relative importance that patients and caregivers attach to each indicator and the incremental value of improvements across performance levels, acknowledging that expectations for high-quality dying are nonlinear and that each indicator contributes differently to perceived quality. Weighted scores were rescaled to a 0–100 range for interpretability (8, 25).

Although the previous study found broadly consistent EOL care priorities across countries, potential cross-national variation in indicator weights cannot be ruled out (8, 26). To ensure robustness, we conducted a sensitivity analysis using unweighted simple-sum scores as the dependent variable when examining associated factors and training effects. Results were consistent with those obtained using weighted scores (see Supplementary Tables 2, 4).

In this study, respondents completed the translated Chinese version of the QODDI, which had previously undergone systematic translation, cultural adaptation, and psychometric validation among palliative and hospice care professionals in Shanghai. Prior testing demonstrated sound psychometric properties, including satisfactory item performance, high internal consistency, strong test–retest reliability, and good convergent, divergent, and concurrent validity (27). In the present sample, Cronbach’s *α* was 0.9615, indicating high internal consistency among Chinese healthcare professionals nationwide (see Tables 1, 2).

**Table 1:** Sample characteristics.

Variable	Total (*N* = 2,465)
Institutional background	
Funding source of the healthcare institution	
Public	2,401 (97.4%)
Private	64 (2.6%)
Type of healthcare institution	
Tertiary/secondary/specialized hospital	959 (38.9%)
Community healthcare center	1,213 (49.2%)
Other types of institutions	293 (11.9%)
Multidisciplinary end-of-life service team	
Yes	1720 (69.8%)
No	745 (30.2%)
Sociodemographic Information	
Gender	
Male	230 (9.3%)
Female	2,235 (90.7%)
Birth cohort	
70s	462 (18.7%)
80s	1,098 (44.5%)
90s	750 (30.4%)
00s	155 (6.3%)
Educational level	
High school and below	16 (0.6%)
Junior college degree	243 (9.9%)
Bachelor’s degree	2056 (83.4%)
Master’s degree and above	150 (6.1%)
Religious belief	
Yes	117 (4.7%)
No	2,348 (95.3%)
Occupational data	
Occupational category	
Physician	578 (24.4%)
Nurse	1756 (74.2%)
Social worker	33 (1.4%)
Professional title	
Senior	74 (3.0%)
Associate senior	360 (14.6%)
Intermediate	1,327 (53.8%)
Junior	655 (26.6%)
None	49 (2.0%)
Average survival period of patients served	
Less than 1 month	324 (13.1%)
1–3 months	894 (36.3%)
3–6 months	1,110 (45.0%)
6 months or more	137 (5.6%)
Years of service in palliative and hospice care	
Mean (SD)	3.93 (4.15)
Number of patients served	
Mean (SD)	194.43 (1444.98)

**Table 2:** Weighted quality of death and dying statistics.

Questionnaires	Labels	Mean (SD)
Q1. The places where healthcare providers treated patient were clean, safe, and comfortable	Clean and safe space	0.89 (0.40)
Q2. Patient was able to be cared for and die at place of choice	Preferred place of death	0.49 (0.27)
Q3. Healthcare providers provided appropriate levels and quality of life extending treatments	Quality of life extending treatments	0.83 (0.39)
Q4. Healthcare professionals supported patient’s spiritual, religious, and cultural needs	Spiritual needs	0.39 (0.21)
Q5. Care was well coordinated across different healthcare providers	Care was well coordinated	0.61 (0.26)
Q6. Healthcare providers controlled pain and discomfort to patient’s desired levels	Managed pain and discomfort	0.97 (0.41)
Q7. Healthcare providers helped patient cope emotionally	Cope emotionally	0.61 (0.25)
Q8. Healthcare providers encouraged contact with friends and family	Contact with family	0.49 (0.20)
Q9. Healthcare providers helped with patient’s nonmedical concerns	Non-medical concerns	0.34 (0.18)
Q10. Healthcare providers delivered clear and timely information so patients could make informed decisions	Clear and timely information	0.76 (0.33)
Q11. Healthcare providers asked enough questions to understand patient’s needs	Asked enough questions	0.68 (0.31)
Q12. Healthcare providers mostly treated patients kindly and sympathetically	Treated kindly	0.89 (0.35)
Q13. Costs were not a barrier to patient getting appropriate care	Costs were not a barrier	0.48 (0.27)
Total score of weighted quality of death and dying index		8.43 (3.31)
Rescaled quality of death and dying index (0–100)		90.55 (12.81)

#### 2.2.2 Institutional, sociodemographic, and occupational data

Institutional background information covered the funding source of the institution, types of institutions, and whether the institution has established a multidisciplinary end-of-life service team. Sociodemographic information collected included gender, birth cohort, educational level, and religious beliefs. Occupational data included occupational category, professional title, years of service in palliative care, the total number of patients served, and the average survival period of patients served.

#### 2.2.3 Training experiences in palliative and hospice care

Participants were asked a binary question to denote whether they had received municipal or district-level (or above) palliative and hospice care-related training. This variable was coded as 0 (not trained) or 1 (trained).

### 2.3 Data analysis

Linear regression models were employed to examine the influence of individual and institutional characteristics of healthcare professionals on Quality of Death and Dying Index assessments. Given regional disparities in palliative care infrastructure, clustered standard errors were utilized to address heteroscedasticity and obtain unbiased coefficients, with cluster groups delineated according to China’s provinces (28).

Subsequently, the study evaluated whether the recent widespread palliative care training programs improved QODDI scores. A propensity score matching analysis was conducted to accurately capture the potential intervention effect of training on quality rating by using STATA’s community-contributed command PSMATCH2 (29). This quasi-experimental method balanced the sample between healthcare professionals who had received palliative care training and those who had not, reducing potential selection bias and confounding effects. Given our large sample size, we employed the most common 1:1 nearest neighbor matching technique without replacement (30, 31). The propensity scores were calculated based on the aforementioned socio-demographic, occupational, and institutional background variables. To further ensure the robustness of our findings, we additionally applied a kernel density matching method with a bandwidth of 0.2 and re-estimated the models. The results remained consistent with those from the main analysis (see Supplementary Table 4).

All analyses were performed using STATA 17 BE (32).

## 3 Results

### 3.1 Sample characteristics

The sample included 2,465 respondents, primarily from publicly funded healthcare institutions (2,401 [97.4%]), with a small proportion from privately funded institutions (64 [2.6%]). Respondents were mostly affiliated with community healthcare centers (1,213 [49.2%]), followed by tertiary, secondary, or specialized hospitals (959 [38.9%]), and other institutions (293 [11.9%]). A majority (1,720 [69.8%]) reported the establishment of an end-of-life service team at their institution.

The sample was predominantly female (2,235 [90.7%]) and included respondents born in the 1970s (462 [18.7%]), 1980s (1,098 [44.5%]), 1990s (750 [30.4%]), and 2000s (155 [6.3%]). Most held a bachelor’s degree (2,056 [83.4%]), with others holding junior college degrees (243 [9.9%]), master’s degrees or higher (150 [6.1%]), and high school or below (16 [0.6%]). The majority (2,348 [95.3%]) reported no religious affiliation.

Occupationally, 1,756 (74.2%) were nurses, 578 (24.4%) were physicians, and 33 (1.4%) were social workers. Professional titles varied, with 1,327 (53.8%) holding intermediate titles, 655 (26.6%) junior titles, 360 (14.6%) associate senior titles, 74 (3.0%) senior titles, and 49 (2.0%) without titles. The average survival period of patients served was 3–6 months for 1,110 (45.0%), 1–3 months for 894 (36.3%), under 1 month for 324 (13.1%), and 6 months or more for 137 (5.6%). Respondents had an average (SD) of 3.93 years (4.15) of service in palliative and hospice care, with a mean (SD) of 194.43 (1444.98) patients cared for over their careers.

### 3.2 Healthcare professionals’ ratings on quality of death and dying index

The mean (SD) weighted Index score was 8.43 (3.31), with a rescaled score of 90.55 (12.81) on a 0–100 scale. Chinese healthcare providers rated highly for controlling pain and discomfort (Mean [SD], 0.97 [0.41]). Meanwhile, lower ratings were observed in areas such as supporting patients’ spiritual, religious, and cultural needs (0.39 [0.21]), addressing nonmedical concerns (0.34 [0.18]), and ensuring that costs were not a barrier to receiving appropriate care (0.48 [0.27]).

### 3.3 Factors influencing quality of death and dying index ratings

The linear regression analysis examined various factors influencing quality ratings among healthcare professionals (*N* = 2,366). The results in Table 3 revealed several significant predictors.

**Table 3:** Factors influencing quality of death and dying index rating (*N* = 2,366).

Variable	Coef.	SE	95% CI
Funding source of healthcare institution^a^			
Private	−0.531	1.54	[−3.68, 2.61]
Type of healthcare institution^b^			
Community healthcare center	−0.412	0.87	[−2.19, 1.37]
Other types of institutions	−0.367	0.80	[−1.99, 1.26]
Multidisciplinary end-of-life service team^c^			
Yes	3.441^***^	0.79	[1.83, 5.05]
Gender^d^			
Female	2.110^**^	0.59	[0.90, 3.32]
Birth cohort^e^			
80s	−1.444	0.92	[−3.33, 0.44]
90s	−2.907^**^	0.95	[−4.85, −0.96]
00s	−3.995^**^	1.33	[−6.71, −1.28]
Educational level^f^			
Junior college degree	5.383^**^	1.85	[1.60, 9.16]
Bachelor’s degree	5.261^*^	2.44	[0.27, 10.25]
Master’s degree and above	3.775	2.87	[−2.10, 9.65]
Religious belief^g^			
No	0.632	1.00	[−1.40, 2.67]
Occupational category^h^			
Nurse	0.196	1.22	[−2.31, 2.70]
Social worker	2.277	2.62	[−3.07, 7.63]
Professional title^i^			
Associate senior	1.837	2.00	[−2.26, 5.93]
Intermediate	1.323	1.82	[−2.40, 5.05]
Junior	1.824	2.25	[−2.77, 6.42]
None	−2.690	3.51	[−9.88, 4.50]
Average survival period of patients served^j^			
1–3 months	1.333	0.96	[−0.63, 3.30]
3–6 months	2.141^**^	0.61	[0.90, 3.38]
6 months or more	1.862	1.08	[−0.35, 4.08]
Years in palliative and hospice care	0.203^**^	0.07	[0.06, 0.35]
Number of patients served	0.000172	0.00	[−0.00, 0.00]

***p < 0.001, **p < 0.01, *p < 0.05; reference groups: ^a^ Public; ^b^ Tertiary/secondary/specialized hospital; ^c^ No; ^d^ Male; ^e^ 70s; ^f^ High school and below; ^g^ Yes; ^h^ Physician; ^i^ Senior; ^j^ Less than 1 month.

The establishment of a multidisciplinary team within an institution was positively associated with higher ratings (*β* = 3.441, SE = 0.79, *p* = 0.000).

Gender was also a significant factor, with female healthcare professionals rating 2.110 points higher than their male counterparts (SE = 0.59, *p* = 0.001). Regarding birth cohort, medical personnel born in the 1990s (*β* = −2.907, SE = 0.95, *p* = 0.005) and 2000s (*β* = −3.995, SE = 1.33, *p* = 0.005) provided significantly lower ratings compared to those born in the 1970s. Additionally, respondents holding a junior college degree (β = 5.383, SE = 1.85, *p* = 0.007) or a bachelor’s degree (β = 5.261, SE = 2.44, *p* = 0.039) reported higher ratings compared to those who received a high school education or below.

Occupational experience also influenced ratings on the Quality of Death and Dying Index. Healthcare professionals serving patients with a survival period of 3–6 months rated 2.14 points higher (SE = 0.61, *p* = 0.001) compared to those serving patients with less than one month of survival. Years of service in palliative and hospice care positively influenced ratings (*β* = 0.203, SE = 0.07, *p* = 0.008), indicating that greater experience in this field correlates with higher perceived quality of death and dying.

In contrast, institutional funding sources, types of healthcare institutions, whether having religious beliefs, occupational categories, professional titles, and the number of patients served did not significantly influence ratings.

### 3.4 Effect of training experiences on quality of death and dying index ratings

We further examined whether participation in hospice and palliative care training programs influenced healthcare professionals’ QODDI ratings. Given that institutional, individual, and occupational characteristics might affect the likelihood of participating in these programs (24), a propensity score matching approach was adopted to minimize selection bias rather than including training directly as a covariate in the regression model. The matching process effectively balanced the mean values of key covariates between the trained and untrained groups, with standardized bias reduced to below the 5 percent threshold for most variables.

**Figure 1:**
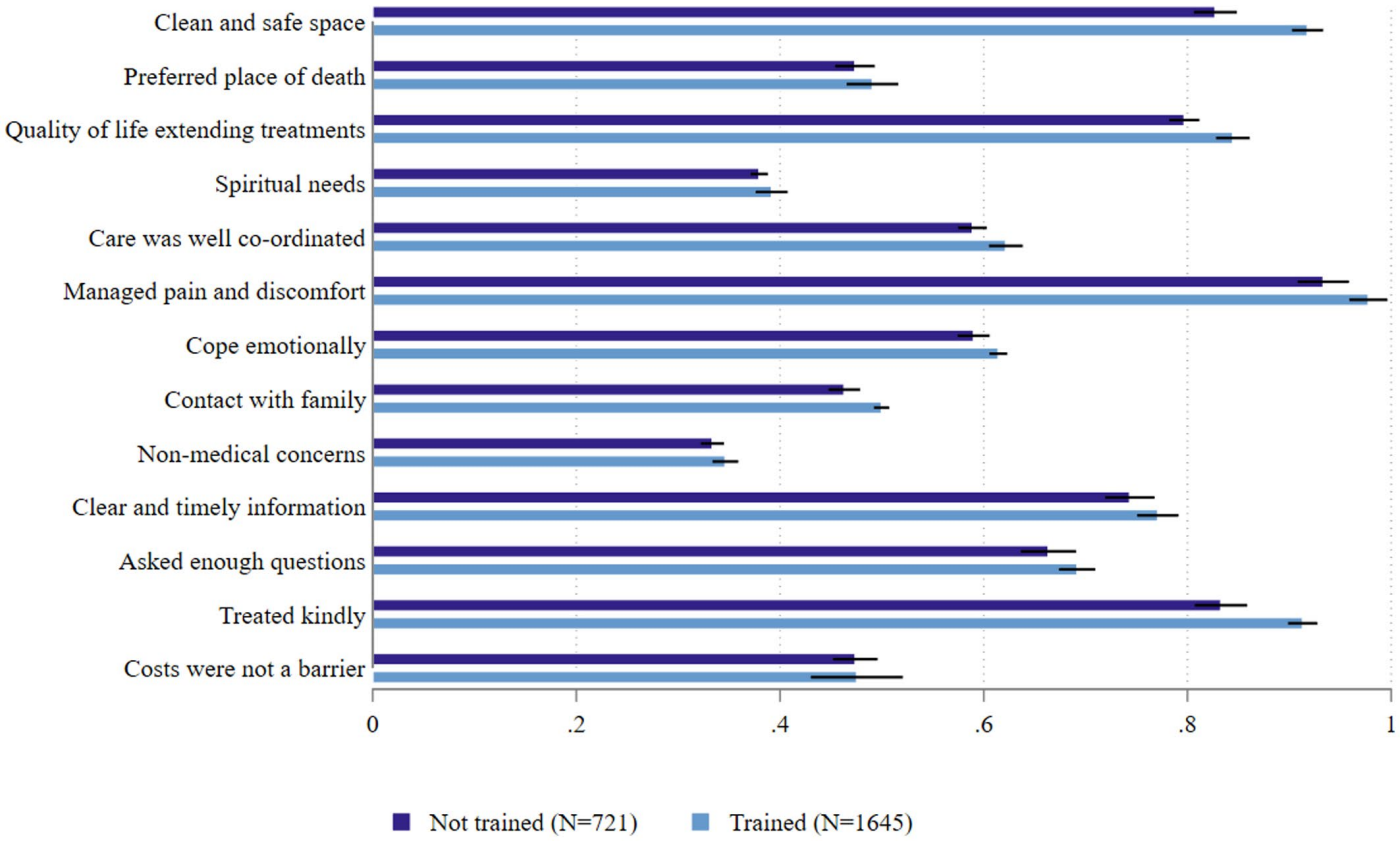
The mean and confidence intervals of Chinese healthcare professionals’ ratings on 13 QODDI attributes after matching.

Figure 1 illustrates the means and confidence intervals for each Index attribute after matching. Overall, healthcare professionals with training reported higher average scores across all questions. Notably, significant improvements were observed in the following areas: clean and safe space (*p* = 0.022), care coordination (*p* = 0.046), contact with family (*p* = 0.003), and being treated kindly (*p* = 0.001). Additional t-test results comparing trained and untrained samples before and after matching are provided in Supplementary Table 3.

**Figure 2:**
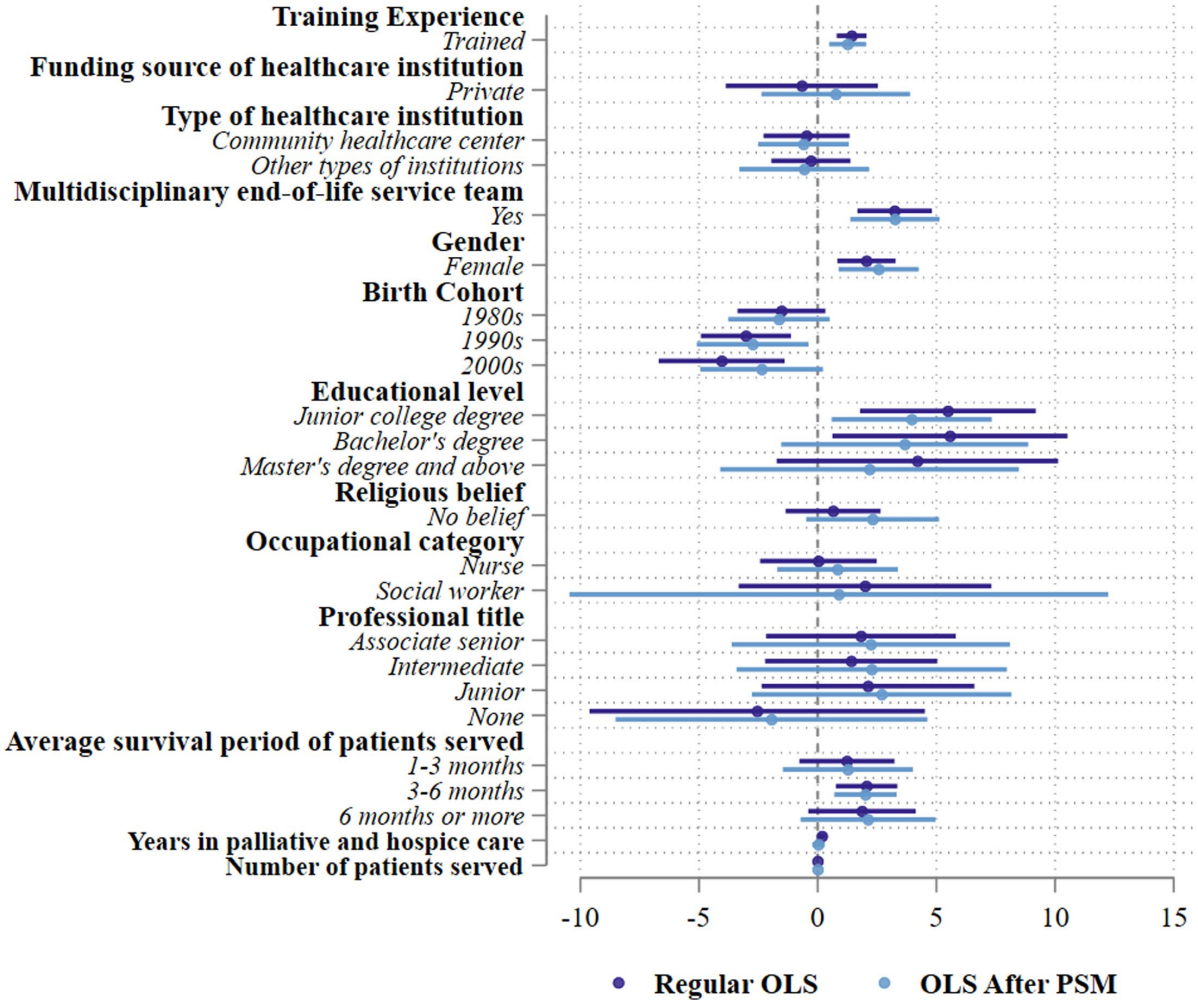
Effect of training experiences, institutional, sociodemographic, and occupational factors, before and after propensity-score-matching.

Figure 2 graphically presents the regression results before and after propensity score matching. Training experience was positively associated with higher QODDI ratings in both models, although the effect size was somewhat reduced after matching. In the multivariate OLS model, professionals who had received palliative care training reported significantly higher quality ratings (*β* = 1.430, SE = 4.65, *p* < 0.001). After matching, the association remained statistically significant (β = 1.262, SE = 3.32, *p* = 0.03), indicating that the beneficial effect of training persisted even after accounting for observable differences between trained and untrained respondents.

Moreover, the overall patterns of significant associations for institutional, demographic, and occupational variables were largely consistent with those reported in Table 3, supporting the robustness of the results after including training as a key influencing factor. Detailed regression coefficients are provided in the Supplementary Table 4 for reference.

## 4 Discussion

This study presents the first large-scale analysis of end-of-life care perceptions across China, involving 2,465 healthcare professionals with palliative and hospice care experience, including doctors, nurses, and medical social workers. It investigates how institutional characteristics, sociodemographic profiles, occupational experiences, and participation in palliative care training influence Quality of Death and Dying Index ratings.

The results show that healthcare professionals in China reported a relatively high average QODDI score, with a rescaled mean of 90.55. However, considerable variation exists within the scores, suggesting internal disparities in the development and quality of end-of-life care across China. This variability calls for caution when drawing international comparisons of QODDI scores, as intranational disparities may skew evaluations (11). Future cross-national surveys should aim to include a more representative sample of Chinese experts to enhance the reliability of comparative analyses.

This study further deepens the understanding of the determinants of QODDI ratings among Chinese healthcare professionals, focusing on institutional, sociodemographic, occupational characteristics, and training experiences.

At the institutional level, the source or type of funding did not significantly affect quality evaluations. This finding is likely explained by the institutional composition of China’s palliative care system, which is predominantly hospital-based. Most end-of-life care is provided by oncology wards in tertiary hospitals or public primary care institutions, while private nursing homes or hospices rarely offer such services (33). In our sample, only 2.6% of respondents came from private facilities, resulting in low variance and limited statistical power to detect differences.

However, the presence of a dedicated palliative care team within an institution was positively associated with higher rating scores. This finding aligns with previous research highlighting that team-based collaboration improves the delivery and continuity of end-of-life care and mitigates professional burnout (34, 35). Although substantial evidence highlights the critical role of multidisciplinary teams in optimizing care quality (36, 37), approximately 30% of the medical institutions surveyed have yet to establish such teams. This proportion is likely even higher in institutions we did not survey. The development of hospice and palliative care in China remains at an early stage. Except for a few major cities, many healthcare institutions lack full multidisciplinary end-of-life care teams or have incomplete team structures. Ideally, a multidisciplinary team should include physicians, nurses, social workers, pharmacists, nutritionists, and volunteers, each contributing distinct expertise (38–40). Nevertheless, current practice remains predominantly physician-led, particularly in tertiary hospitals, where doctors often focus on disease treatment while offering limited attention to psychological, social, or spiritual dimensions of care (41). Moreover, insufficient training in communication and medical humanities further constrains holistic end-of-life service delivery (42, 43). Therefore, to improve the quality of death and dying in China, policy efforts should prioritize increasing financial and human capital investment, strengthening interprofessional collaboration, and ensuring equal participation of all team members in care decisions.

Gender, birth cohort, and educational level emerged as key predictors of index ratings. Female professionals tended to assign higher scores, consistent with prior research (22, 44). From a gender role perspective, women are socialized to value emotional labor, empathy, and relational communication (45), which align with the ethos of palliative care. Female healthcare workers, especially nurses and social workers, may be more attuned to patients’ psychological and family needs and more sensitive to the emotional dimensions of dying (22, 46). Cultural sociology also suggests that gendered expectations of caregiving in Chinese society shape professional attitudes, potentially leading female practitioners to perceive a higher quality of end-of-life care (45, 47). Future research should further examine how social norms and gendered divisions of care influence professionals’ understandings of a “good death”.

Generational differences also emerged. Younger healthcare professionals, particularly those born in the 1990s and 2000s, provided lower ratings. This trend may be due to limited death-related education in training for younger generations (48), coupled with less personal experience with death in daily and clinical settings (49). Furthermore, selection bias might play a role, where older professionals who continue to work in palliative care may inherently have a stronger commitment to their work. More detailed investigations are needed to differentiate these generational effects on quality ratings.

While higher educational levels were generally associated with higher rating scores, this trend did not hold for individuals with graduate degrees or above. This discrepancy could be due to the diverse career paths and specializations pursued by highly educated professionals, which might dilute the focus on palliative care. For instance, medical doctors with advanced degrees often emphasize treatment, whereas nurses engage more closely in direct caregiving and patient interaction in later stages, potentially shaping distinct perceptions of death and dying (44). Additionally, other research suggests that health and death literacy, rather than formal education, may be the stronger predictor of quality ratings (50).

These findings underscore the importance of tailoring training and policy initiatives to diverse demographic groups. Encouraging greater participation of male professionals and physicians in palliative care through gender-sensitive strategies could enhance engagement and empathy. Expanding educational opportunities for younger practitioners and providing financial or institutional support may help cultivate early-career commitment. Further studies should also explore how highly educated professionals conceptualize “quality death” to inform the design of advanced training curricula.

Occupational experience also influenced QODDI ratings, with healthcare providers caring for patients expected to survive 3–6 months rating care quality higher than those working with patients in very late (≤1 month) or extended (≥6 months) stages. This result likely reflects nuanced differences in care focus: the 3–6 month period often allows for stable, comprehensive palliative interventions, enabling providers to engage in holistic care, including psychological support and family counseling (51). In contrast, imminent end-of-life situations prioritize symptom management under time pressure, while longer prognoses can create uncertainty between curative and palliative goals, potentially diluting end-of-life quality considerations (52, 53).

Additionally, the longer the healthcare professionals had worked in palliative and hospice care, the higher their ratings were. This result suggests that accumulated experience enhances perceived quality, potentially due to increased expertise and comfort in dealing with end-of-life issues over time (19, 54).

Propensity score matching analyses further confirmed that participation in palliative and hospice care training programs was associated with higher QODDI scores. This finding aligns with prior evidence that continuing education improves professionals’ comfort and efficacy in end-of-life care delivery (23, 55). In China, formal death-related education in medical and nursing schools remains limited, and death is often considered a culturally sensitive or taboo topic. Consequently, both novice and experienced practitioners may experience anxiety and discomfort when caring for dying patients (22). Training programs thus serve as crucial compensatory mechanisms to address knowledge and attitudinal gaps. However, in this study, the difference in ratings between trained and untrained groups, while significant, was not large, potentially because we did not distinguish between short-term and long-term training formats. Previous research indicates that while brief training experiences can increase knowledge and self-efficacy, transforming practice behavior requires ongoing education and experiential learning (56–58). Therefore, longitudinal studies are needed to track the sustained impact of such training initiatives on quality of death and dying scoring. Meanwhile, from a policy perspective, it is necessary to expand the reach and duration of training, integrate end-of-life communication and humanistic care into medical education, and establish standardized curricula to maximize behavioral changes among practitioners.

It also needs to be noted that, while QODDI provides a comprehensive framework, the meaning of a “good death” is deeply embedded in cultural norms (59). In East Asian societies, including China, Japan, and Korea, collectivist and family-centered values shape distinct attitudes toward dying compared with Western societies (60). In the Chinese context, concepts such as filial piety, gratitude expression, and family harmony strongly influence end-of-life experiences. Disclosure of prognosis, truth-telling, and patient autonomy often remain family-mediated decisions. Traditional beliefs and rituals surrounding death further shape healthcare professionals’ attitudes and practices (26, 60–62). These cultural particularities underscore the need to contextualize QODDI assessments and to develop culturally sensitive measures that reflect local understandings of dignity, family connectedness, and spiritual peace (1). Increasingly, Chinese scholars have begun to explore culturally grounded frameworks of a “good death,” calling for policy, legal, and institutional reforms to align end-of-life care with local values (63, 64).

Despite being the first large-scale empirical study in China to explore the factors influencing inconsistent evaluations of death and dying quality by end-of-life care professionals, this study has several limitations that warrant consideration. First, the data were not collected using random sampling, which could introduce statistical bias. Second, the reliance on self-reported data may have introduced social desirability bias, as respondents often gave high ratings that could reflect professional expectations or institutional norms rather than objective evaluations. Third, the cross-sectional design of the study limits the ability to infer causality or track changes over time. All associations identified should be interpreted as correlational rather than causal. Future research could adopt more rigorous designs, including longitudinal follow-ups, experimental, or quasi-experimental approaches, to provide stronger evidence regarding the causal effects of training interventions and institutional characteristics. Lastly, the calculation of Index scores used preference weights offered by the Lien Foundation researchers. Given cultural differences, the appropriateness of these weights in the Chinese context warrants further scrutiny.

## 5 Conclusion

This study identified several factors influencing Chinese healthcare professionals’ evaluations of the quality of death and dying. Higher ratings were associated with being female, born in the 1970s, holding a bachelor’s degree, working in institutions with multidisciplinary teams, having longer years of service, and receiving hospice and palliative care training. Strengthening multidisciplinary cooperation, improving professional training, and promoting gender- and age-sensitive recruitment and education strategies could contribute to better end-of-life care, particularly as population aging increases and the demand for quality dying experiences grows. These findings may inform national interventions to promote equitable and person-centered end-of-life care in China.

## Data Availability

The datasets presented in this article are not readily available because they are kept confidential to ensure participant anonymity. The author can share further findings and secondary data products on reasonable requests. Requests to access the datasets should be directed to chengmmthu@163.com.
