# Supplementary material for: Factors influencing healthcare professionals’ rating on quality of death and dying: a nationwide cross-sectional study in China
Source: Front Public Health. 2025 Nov 20;13:1722430. doi: 10.3389/fpubh.2025.1722430 (PMC12677404; doi:10.3389/fpubh.2025.1722430)
Supplement: Supplementary file 1 [file Supplementary_file_1.docx]

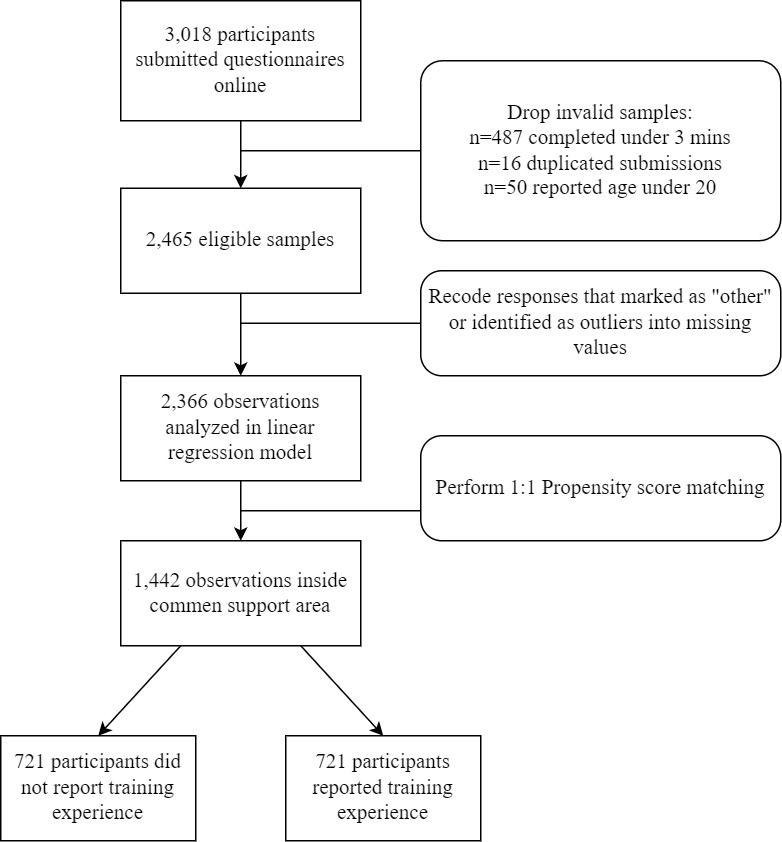


### **Supplementary Figure 1. Flowchart of sample selection process**

### **Supplementary Table 2. Factors influencing Quality of Death and Dying Index rating, using unweighted score (N=2,366)**

|  | Coef. | SE | 95% CI |
| --- | --- | --- | --- |
| Funding source of healthcare institution ^a^ |  |  |  |
| *Private* | -0.52 | 0.94 | [-2.45,1.41] |
| Type of healthcare institution ^b^ |  |  |  |
| *Community healthcare center* | -0.38 | 0.71 | [-1.83,1.08] |
| *Other types of institutions* | -0.02 | 0.66 | [-1.37,1.32] |
| Multidisciplinary end-of-life service team ^c^ |  |  |  |
| *Yes* | 3.24*** | 0.67 | [1.86,4.62] |
| Gender ^d^ |  |  |  |
| *Female* | 1.16** | 0.34 | [0.47,1.85] |
| Birth cohort ^e^ |  |  |  |
| *80s* | -1.43 | 0.72 | [-2.91,0.04] |
| *90s* | -2.62** | 0.76 | [-4.18,-1.07] |
| *00s* | -3.73*** | 0.94 | [-5.66,-1.81] |
| Educational level ^f^ |  |  |  |
| *Junior college degree* | 4.22** | 1.21 | [1.75,6.69] |
| *Bachelor's degree* | 4.44* | 1.76 | [0.85,8.04] |
| *Master's degree and above* | 5.01* | 1.86 | [1.22,8.81] |
| Religious belief ^g^ |  |  |  |
| *No* | 0.30 | 0.62 | [-0.96,1.57] |
| Occupational category ^h^ |  |  |  |
| *Nurse* | 0.31 | 1.00 | [-1.73,2.35] |
| *Social worker* | 1.40 | 2.19 | [-3.09,5.89] |
| Professional title ^i^ |  |  |  |
| *Associate senior* | 1.29 | 1.33 | [-1.42,4] |
| *Intermediate* | 1.12 | 1.18 | [-1.29,3.52] |
| *Junior* | 1.57 | 1.55 | [-1.61,4.75] |
| *None* | -0.82 | 2.03 | [-4.97,3.33] |
| Average survival period of patients served ^j^ |  |  |  |
| *1-3 months* | 0.85 | 0.70 | [-0.57,2.27] |
| *3-6 months* | 1.45** | 0.42 | [0.6,2.31] |
| *6 months or more* | 1.01 | 0.66 | [-0.33,2.35] |
| Years in palliative and hospice care | 0.19*** | 0.05 | [0.08,0.29] |
| Number of patients served | 0.00 | 0.00 | [0,0] |

Notes: ***p<0.001,**P<0.01,*p<0.05; Reference groups: a - Public; b - Tertiary/ secondary/ specialized hospital; c - No; d -Male; e - 70s; f - High school and below; g - Yes; h - Physician; i - Senior; j - Less than 1 month.

### **Supplementary Table 3. Comparison of QODDI ratings, before and after propensity score-matching**

| \|  \| Before Matching \| \| \| \| After Matching \| \| \| \| \| --- \| --- \| --- \| --- \| --- \| --- \| --- \| --- \| --- \| \|  \| Total  (N = 2465) \| Not trained (N = 721) \| Trained  (N = 1682) \| p-value \| Total  (N = 1442) \| Not trained  (N = 721) \| Trained  (N = 721) \| p-value \| \| **Clean and safe space** \|  \|  \|  \| <0.001 \|  \|  \|  \| 0.022 \| \| Mean (SD) \| 0.89 (0.40) \| 0.83 (0.47) \| 0.92 (0.36) \|  \| 0.85 (0.43) \| 0.83 (0.46) \| 0.88 (0.39) \|  \| \| **Preferred place of death** \|  \|  \|  \| 0.049 \|  \|  \|  \| 0.119 \| \| Mean (SD) \| 0.49 (0.27) \| 0.47 (0.31) \| 0.49 (0.26) \|  \| 0.48 (0.28) \| 0.47 (0.30) \| 0.49 (0.26) \|  \| \| **Quality of life extending treatments** \|  \|  \|  \| 0.004 \|  \|  \|  \| 0.385 \| \| Mean (SD) \| 0.83 (0.39) \| 0.80 (0.43) \| 0.85 (0.37) \|  \| 0.81 (0.41) \| 0.80 (0.42) \| 0.82 (0.39) \|  \| \| **Spiritual needs** \|  \|  \|  \| 0.089 \|  \|  \|  \| 0.430 \| \| Mean (SD) \| 0.39 (0.21) \| 0.38 (0.23) \| 0.39 (0.20) \|  \| 0.38 (0.22) \| 0.38 (0.23) \| 0.39 (0.21) \|  \| \| **Care was well co-ordinated** \|  \|  \|  \| 0.001 \|  \|  \|  \| 0.046 \| \| Mean (SD) \| 0.61 (0.26) \| 0.59 (0.30) \| 0.62 (0.24) \|  \| 0.60 (0.27) \| 0.59 (0.29) \| 0.61 (0.26) \|  \| \| **Managed pain and discomfort** \|  \|  \|  \| 0.005 \|  \|  \|  \| 0.102 \| \| Mean (SD) \| 0.97 (0.41) \| 0.93 (0.47) \| 0.98 (0.37) \|  \| 0.95 (0.42) \| 0.93 (0.45) \| 0.97 (0.38) \|  \| \| **Cope emotionally** \|  \|  \|  \| 0.011 \|  \|  \|  \| 0.110 \| \| Mean (SD) \| 0.61 (0.25) \| 0.59 (0.29) \| 0.62 (0.22) \|  \| 0.60 (0.25) \| 0.59 (0.28) \| 0.61 (0.22) \|  \| \| **Contact with family** \|  \|  \|  \| <0.001 \|  \|  \|  \| 0.003 \| \| Mean (SD) \| 0.49 (0.20) \| 0.46 (0.23) \| 0.50 (0.18) \|  \| 0.48 (0.20) \| 0.46 (0.23) \| 0.49 (0.18) \|  \| \| **Non-medical concerns** \|  \|  \|  \| 0.048 \|  \|  \|  \| 0.259 \| \| Mean (SD) \| 0.34 (0.18) \| 0.34 (0.21) \| 0.35 (0.17) \|  \| 0.34 (0.19) \| 0.33 (0.21) \| 0.34 (0.18) \|  \| \| **Clear and timely information** \|  \|  \|  \| 0.020 \|  \|  \|  \| 0.066 \| \| Mean (SD) \| 0.76 (0.33) \| 0.74 (0.39) \| 0.77 (0.30) \|  \| 0.76 (0.35) \| 0.74 (0.38) \| 0.77 (0.31) \|  \| \| **Asked enough questions** \|  \|  \|  \| 0.014 \|  \|  \|  \| 0.125 \| \| Mean (SD) \| 0.68 (0.31) \| 0.67 (0.36) \| 0.69 (0.28) \|  \| 0.67 (0.33) \| 0.66 (0.35) \| 0.68 (0.30) \|  \| \| **Treated kindly** \|  \|  \|  \| <0.001 \|  \|  \|  \| 0.001 \| \| Mean (SD) \| 0.89 (0.35) \| 0.83 (0.42) \| 0.92 (0.31) \|  \| 0.86 (0.37) \| 0.83 (0.41) \| 0.89 (0.32) \|  \| \| **Costs were not a barrier** \|  \|  \|  \| 0.484 \|  \|  \|  \| 0.668 \| \| Mean (SD) \| 0.48 (0.27) \| 0.48 (0.27) \| 0.48 (0.27) \|  \| 0.48 (0.27) \| 0.47 (0.27) \| 0.48 (0.27) \|  \| \| **Total score of weighted QODDI** \|  \|  \|  \| 0.001 \|  \|  \|  \| 0.073 \| \| Mean (SD) \| 8.43 (3.31) \| 8.11 (3.97) \| 8.58 (2.93) \|  \| 8.26 (3.49) \| 8.10 (3.82) \| 8.43 (3.12) \|  \| \| **Rescaled QODDI (0-100)** \|  \|  \|  \| 0.001 \|  \|  \|  \| 0.073 \| \| Mean (SD) \| 90.55 (12.81) \| 89.30 (15.40) \| 91.14 (11.37) \|  \| 89.91 (13.54) \| 89.27 (14.81) \| 90.55 (12.10) \|  \| |
| --- | --- | --- | --- | --- | --- | --- | --- | --- | --- | --- | --- | --- | --- | --- | --- | --- | --- | --- | --- | --- | --- | --- | --- | --- | --- | --- | --- | --- | --- | --- | --- | --- | --- | --- | --- | --- | --- | --- | --- | --- | --- | --- | --- | --- | --- | --- | --- | --- | --- | --- | --- | --- | --- | --- | --- | --- | --- | --- | --- | --- | --- | --- | --- | --- | --- | --- | --- | --- | --- | --- | --- | --- | --- | --- | --- | --- | --- | --- | --- | --- | --- | --- | --- | --- | --- | --- | --- | --- | --- | --- | --- | --- | --- | --- | --- | --- | --- | --- | --- | --- | --- | --- | --- | --- | --- | --- | --- | --- | --- | --- | --- | --- | --- | --- | --- | --- | --- | --- | --- | --- | --- | --- | --- | --- | --- | --- | --- | --- | --- | --- | --- | --- | --- | --- | --- | --- | --- | --- | --- | --- | --- | --- | --- | --- | --- | --- | --- | --- | --- | --- | --- | --- | --- | --- | --- | --- | --- | --- | --- | --- | --- | --- | --- | --- | --- | --- | --- | --- | --- | --- | --- | --- | --- | --- | --- | --- | --- | --- | --- | --- | --- | --- | --- | --- | --- | --- | --- | --- | --- | --- | --- | --- | --- | --- | --- | --- | --- | --- | --- | --- | --- | --- | --- | --- | --- | --- | --- | --- | --- | --- | --- | --- | --- | --- | --- | --- | --- | --- | --- | --- | --- | --- | --- | --- | --- | --- | --- | --- | --- | --- | --- | --- | --- | --- | --- | --- | --- | --- | --- | --- | --- | --- | --- | --- | --- | --- | --- | --- | --- | --- | --- | --- | --- | --- | --- | --- | --- | --- | --- | --- | --- | --- | --- | --- | --- | --- | --- | --- | --- | --- | --- | --- | --- | --- | --- | --- | --- | --- | --- | --- | --- | --- | --- | --- | --- | --- | --- | --- |

**Supplementary Table 4. Effect of training experiences, before and after propensity-score-matching**

|  | **Model 1** | | | | |  | **Model 2** | | | | | | | | | |  | | **Model 3** | | | | | | | | | |  |
| --- | --- | --- | --- | --- | --- | --- | --- | --- | --- | --- | --- | --- | --- | --- | --- | --- | --- | --- | --- | --- | --- | --- | --- | --- | --- | --- | --- | --- | --- |
|  | OLS  (N=2,366) | |  | OLS after PSM  (N=1,442) | |  | | OLS  (N=2,366) | | | |  | | OLS after PSM  (N=1,442) | | | |  | | OLS  (N=2,366) | | | |  | | OLS after PSM  (N=2,366) | | | |
|  | Coef. | SE |  | Coef. | SE |  | | Coef. | | SE | |  | | Coef. | | SE | |  | |  | |  | |  | |  | |  | |
| Training Experience |  |  |  |  |  |  |  | |  | |  | |  | |  | |  | |  | |  | |  | |  | |  | |  |
| *Yes* | 1.430^***^ | (4.65) |  | 1.262^**^ | (3.32) |  | 0.939*** | | (3.87) | |  | | 0.842** | | (2.77) | |  | | 1.430^***^ | | (4.65) | |  | | 1.481^**^ | | (3.43) | |  |
| Funding source of healthcare institution ^a^ |  |  |  |  |  |  |  | |  | |  | |  | |  | |  | |  | |  | |  | |  | |  | |  |
| *Private* | -0.669 | (-0.43) |  | 0.760 | (0.50) |  | -0.613 | | (-0.64) | |  | | 0.161 | | (0.12) | |  | | -0.669 | | (-0.43) | |  | | -0.891 | | (-0.67) | |  |
| Type of healthcare institution ^b^ |  |  |  |  |  |  |  | |  | |  | |  | |  | |  | |  | |  | |  | |  | |  | |  |
| *Community healthcare center* | -0.468 | (-0.53) |  | -0.601 | (-0.64) |  | -0.414 | | (-0.58) | |  | | -0.615 | | (-0.85) | |  | | -0.468 | | (-0.53) | |  | | -0.285 | | (-0.32) | |  |
| *Other types of institutions* | -0.289 | (-0.35) |  | -0.569 | (-0.42) |  | 0.0287 | | (0.04) | |  | | -0.148 | | (-0.14) | |  | | -0.289 | | (-0.35) | |  | | -1.038 | | (-0.71) | |  |
| Multidisciplinary end-of-life service team ^c^ |  |  |  |  |  |  |  | |  | |  | |  | |  | |  | |  | |  | |  | |  | |  | |  |
| *Yes* | 3.240^***^ | (4.22) |  | 3.256^**^ | (3.55) |  | 3.108*** | | (4.69) | |  | | 3.204*** | | (3.91) | |  | | 3.240^***^ | | (4.22) | |  | | 3.408^***^ | | (5.63) | |  |
| Gender ^d^ |  |  |  |  |  |  |  | |  | |  | |  | |  | |  | |  | |  | |  | |  | |  | |  |
| *Female* | 2.054^**^ | (3.42) |  | 2.567^**^ | (3.11) |  | 1.124** | | (3.27) | |  | | 1.264** | | (2.96) | |  | | 2.054^**^ | | (3.42) | |  | | 3.276^***^ | | (4.38) | |  |
| Birth cohort ^e^ |  |  |  |  |  |  |  | |  | |  | |  | |  | |  | |  | |  | |  | |  | |  | |  |
| *80s* | -1.527 | (-1.69) |  | -1.631 | (-1.56) |  | -1.488* | | (-2.09) | |  | | -1.598 | | (-1.97) | |  | | -1.527 | | (-1.69) | |  | | -0.514 | | (-0.46) | |  |
| *90s* | -3.024^**^ | (-3.26) |  | -2.740^*^ | (-2.39) |  | -2.700** | | (-3.63) | |  | | -2.396* | | (-2.69) | |  | | -3.024^**^ | | (-3.26) | |  | | -1.878 | | (-1.68) | |  |
| *00s* | -4.048^**^ | (-3.12) |  | -2.361 | (-1.87) |  | -3.768*** | | (-4.08) | |  | | -2.490* | | (-2.54) | |  | | -4.048^**^ | | (-3.12) | |  | | -2.762^*^ | | (-2.28) | |  |
| Educational level ^f^ |  |  |  |  |  |  |  | |  | |  | |  | |  | |  | |  | |  | |  | |  | |  | |  |
| *Junior college degree* | 5.484^**^ | (3.03) |  | 3.961^*^ | (2.41) |  | 4.286** | | (3.62) | |  | | 2.914* | | (2.64) | |  | | 5.484^**^ | | (3.03) | |  | | 5.829^***^ | | (3.88) | |  |
| *Bachelor's degree* | 5.572^*^ | (2.30) |  | 3.666 | (1.44) |  | 4.648* | | (2.66) | |  | | 2.933 | | (1.58) | |  | | 5.572^*^ | | (2.30) | |  | | 7.150^**^ | | (3.02) | |  |
| *Master's degree and above* | 4.200 | (1.45) |  | 2.182 | (0.71) |  | 5.294** | | (2.83) | |  | | 3.440 | | (1.74) | |  | | 4.200 | | (1.45) | |  | | 2.868 | | (0.88) | |  |
| Religious belief ^g^ |  |  |  |  |  |  |  | |  | |  | |  | |  | |  | |  | |  | |  | |  | |  | |  |
| *No* | 0.647 | (0.66) |  | 2.312 | (1.69) |  | 0.310 | | (0.51) | |  | | 1.814 | | (1.81) | |  | | 0.647 | | (0.66) | |  | | 2.900^*^ | | (2.54) | |  |
| Occupational category ^h^ |  |  |  |  |  |  |  | |  | |  | |  | |  | |  | |  | |  | |  | |  | |  | |  |
| *Nurse* | 0.0301 | (0.03) |  | 0.835 | (0.67) |  | 0.199 | | (0.20) | |  | | 1.131 | | (1.10) | |  | | 0.0301 | | (0.03) | |  | | -0.0352 | | (-0.03) | |  |
| *Social worker* | 1.994 | (0.77) |  | 0.893 | (0.16) |  | 1.212 | | (0.55) | |  | | 0.796 | | (0.21) | |  | | 1.994 | | (0.77) | |  | | 4.274 | | (1.79) | |  |
| Professional title ^i^ |  |  |  |  |  |  |  | |  | |  | |  | |  | |  | |  | |  | |  | |  | |  | |  |
| *Associate senior* | 1.821 | (0.93) |  | 2.238 | (0.78) |  | 1.276 | | (0.98) | |  | | 2.093 | | (0.84) | |  | | 1.821 | | (0.93) | |  | | 2.011 | | (1.01) | |  |
| *Intermediate* | 1.412 | (0.80) |  | 2.275 | (0.82) |  | 1.174 | | (1.02) | |  | | 2.430 | | (1.01) | |  | | 1.412 | | (0.80) | |  | | 1.598 | | (0.85) | |  |
| *Junior* | 2.119 | (0.97) |  | 2.696 | (1.01) |  | 1.766 | | (1.15) | |  | | 2.707 | | (1.17) | |  | | 2.119 | | (0.97) | |  | | 2.773 | | (1.46) | |  |
| *None* | -2.550 | (-0.74) |  | -1.949 | (-0.61) |  | -0.730 | | (-0.37) | |  | | 0.484 | | (0.20) | |  | | -2.550 | | (-0.74) | |  | | -0.544 | | (-0.23) | |  |
| Average survival period of patients served ^j^ |  |  |  |  |  |  |  | |  | |  | |  | |  | |  | |  | |  | |  | |  | |  | |  |
| *1-3 months* | 1.231 | (1.26) |  | 1.269 | (0.95) |  | 0.781 | | (1.10) | |  | | 0.596 | | (0.63) | |  | | 1.231 | | (1.26) | |  | | 1.548 | | (1.76) | |  |
| *3-6 months* | 2.060^**^ | (3.25) |  | 2.012^**^ | (3.14) |  | 1.398** | | (3.28) | |  | | 1.278* | | (2.69) | |  | | 2.060^**^ | | (3.25) | |  | | 1.503 | | (2.01) | |  |
| *6 months or more* | 1.866 | (1.69) |  | 2.119 | (1.53) |  | 1.012 | | (1.52) | |  | | 1.226 | | (1.67) | |  | | 1.866 | | (1.69) | |  | | 0.687 | | (0.39) | |  |
| Years in palliative and hospice care | 0.180^*^ | (2.38) |  | 0.0263 | (0.21) |  | 0.173** | | (3.12) | |  | | 0.0754 | | (0.94) | |  | | 0.180^*^ | | (2.38) | |  | | 0.251^***^ | | (4.51) | |  |
| Number of patients served | 0.000163 | (1.09) |  | 0.00248 | (1.90) |  | 0.000107 | | (0.92) | |  | | 0.00201 | | (1.83) | |  | | 0.000163 | | (1.09) | |  | | 0.000146 | | (0.99) | |  |

Notes: Standard errors are in parentheses; ***p<0.001,**P<0.01,*p<0.05; Reference groups: a - Public; b - Tertiary/ secondary/ specialized hospital; c - No; d -Male; e - 70s; f – High school and below; g - Yes; h - Physician; i - Senior; j - Less than 1 month. Model 1 employs a 1:1 nearest neighbor matching method without replacement, and the corresponding results are illustrated in Figure 2. Model 2 uses the unweighted, cumulative QODDI score as the dependent variable. Model 3 applies kernel density matching with a bandwidth of 0.2 and employs the preference-weighted QODDI score as the dependent variable.
